# Influence of Modified Epoxy Dian Resin on Properties of Nitrile-Butadiene Rubber (NBR)

**DOI:** 10.3390/ma15082766

**Published:** 2022-04-09

**Authors:** Joanna Chudzik, Dariusz M. Bieliński, Yuriy Demchuk, Michael Bratychak, Olena Astakhova

**Affiliations:** 1 Institute of Polymer & Dye Technology, Lodz University of Technology, 12/16 Stefanowskiego Street, 90-537 Lodz, Poland; joanna.chudzik@dokt.p.lodz.pl; 2 Department of Chemical Technology of Oil and Gas Processing, Institute of Chemistry and Chemical Technologies, Lviv Polytechnic National University, 12 Bandery Street, 79013 Lviv, Ukraine; yuriy_demchuk@ukr.net (Y.D.); mbratychak@gmail.com (M.B.); KhTNH.dept@lpnu.ua (O.A.)

**Keywords:** butadiene-acrylonitrile rubber, crosslinking, epoxy resin, modification, mechanical properties, surface energy, adhesion, friction

## Abstract

Due to the increasingly higher requirements for rubber vulcanizates, following the example of previous research on the effect of resin addition on mechanical properties and adhesion of rubbers, the following studies investigated the relationship between the addition of adipic acid-modified epoxy dian resin (ED-24 AK) to butadiene-acrylonitrile rubber (NBR). It can be seen that the addition of ED-24 AK, compared to the reference additive ED-20 (Epidian 5), additionally increase crosslinking density of the system, changes its mechanical and tribological properties, and exerts a positive effect on adhesion of the rubber vulcanizates to glass fiber. ED-20 and ED-24 AK resins do not enter the structure of the vulcanized rubber but act as the additives. ED-20 acts without changes in its structure, and ED-24 AK is a partially crosslinked additive. Modification, especially with ED-24 AK, makes mechanical strength of NBR increased even up to 240% in comparison to virgin rubber vulcanizate. This is responsible for lower friction of the vulcanizates. The decrease in the friction force of NBR due to the modified dian resin addition can reach up to 40%. Adhesion of the modified NBR to glass fibers increases due to its modification with the epoxy resins, however this time the ED-24 is more efficient than ED-24 AK reaching ca. 50% increase comparing to ca. 20% improvement, respectively. The best performance of the resins Generally, the best modification results were obtained when the addition of resins did not exceed 5 phr.

## 1. Introduction

The increasing demands placed on construction materials require the modification of polymers, which is carried out by chemical synthesis [1], physical mixing [2,3], or modification of the surface of finished products [4,5]. In the case of reinforced polymer composites, it becomes of key importance to ensure good adhesion between the continuous and the dispersed phase [6,7,8,9,10,11,12,13,14]. This can be achieved by silanization of the filler particles’ surface [6,7], novolac, and/or urethane aldehyde resin treatment of reinforcing materials based on steel cord or textile fibers [8,9]; admixing thermoplastic resin derived from raw wood rosin [10], melamine resins [11], or low-molecular-weight epoxy resin [12]; litharge and a cobalt salt coating on brass plated steel cords [7]; epoxy matrix modifiers in powder form, including carbon nanotubes, graphene, nanoclay, silica, and natural fillers, improving its adhesion to the layers of reinforcing fibers in the form of laminate fabrics [13]; and polyolefins functionalized with acrylic acid, maleic anhydride, or other thermoplastics with polar groups such as ionomers [14]. One of the possible solutions is admixing of various kinds of adhesive promoters to polymer matrix. This method of pro-adhesive modification is especially popular in rubber technology, making use of silanes [15] or low-molecular-weight polymer resins [16,17], introducing new functional groups, and being able to interact both with polymer and filler/fiber surfaces. It is also known that plasticization of rubber helps to reduce their glass transition temperature [18]. However, due to the fact that traditional plasticizers have limited compatibility with rubber and do not chemically bond with the rubber matrix during vulcanization, there are problems of their migration, extraction upon contact with working media, and volatilization of the plasticizer at elevated temperature. In general, this adversely affects the properties of rubber vulcanizates. In this regard, a modification of NBR (nitrile butadiene rubber) by a reasonably selected brand of epoxy resin that is capable of chemical interactions with rubber macromolecules, such as a non-extractable plasticizer, makes the rubber temperature range of operation expanded. Depending on the type of resin, apart from elasticity and adhesion, it can also modify other properties of the finished product, such as hardness, crosslink density, or tensile strength, allowing other expectations to be met [16,19]. Dian epoxy resin modified with adipic acid (ED-24 AK) contains both carboxyl and epoxy groups [20], which suggests that its addition is likely to have a large impact on the properties of polar polymers such as acrylonitrile-butadiene rubber (NBR).

In general, epoxy resins are widely used as the basis of compositions for the production of, e.g., anti-corrosive coatings and chemically stable adhesives [21,22,23,24], due to their good mechanical, protective, and dielectric properties. Very often, in order to improve their film-forming properties or penetrating ability, facilitating surface modification, epoxy resins are modified with various low-molecular-weight compounds [25,26] and polymers [21]. Compatibility between epoxy resins and macromolecular compounds is very important from the point of view of creating materials exhibiting desired characteristics; therefore, partial replacement of epoxy groups with low-molecular-weight fragments or another compounds, such as organic acids [27], is often practiced. The most frequently used are unsaturated acids, namely acrylic, methacrylic, and crotonic acids. As a result, oligomers devoid of epoxy groups but with double bonds ends are obtained. For this reason, epoxy resins modified with unsaturated acids can find an application in polymer mixes., e.g., ED-24 AK can be used to form crosslink networks, according to the condensation mechanism [20].

The conducted research was aimed at determining the influence of the addition of selected low-molecular-weight epoxy dian resins on the adhesive, mechanical and tribological properties of acrylonitrile-butadiene rubber vulcanizates. Both the effect of the amount of the additive and the modification of the resin with adipic acid were investigated.

## 2. Materials and Methods

### 2.1. Sample Composition

**Figure 1:**
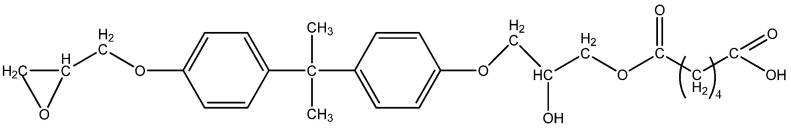
The chemical formula of ED-24AK.

Acrylonitrile-butadiene rubber (NBR; Nancar 2865, Nantex Industry Co., LTD., Kaohsiung, Taiwan) was modified with dian epoxy resin ED-20 (ZChem. CIECH Sarzyna, Nowa Sarzyna, Poland) (Epidian 5) or ED-24 (Epidian 6) modified with adipic acid (ED-24AK). The method of synthesis and characteristics of the resins are described in our previous publication [20]. Briefly, the characteristics of the resins used are as follows:

- ED-20: molecular weight 340 g/mol; epoxy groups content 20%;
- ED-24 AK: molecular weight 970 g/mol; epoxy groups content 4.9%; carboxyl groups content 1.5%;
- ED-24 (Epidian 6) modified with adipic acid (ED-24AK) of the formula is presented in Figure 1.

Sixty grams of Epidian epoxy resin ED-24 and 200 mL of toluene were loaded into a three-necked reactor equipped by a backflow condenser, thermometer, and mechanical stirrer (Expondo, Zielona Góra, Poland). The mixture under constant stirring was heated to 70 °C. Then, 11.4 g of benzyltriethylammonium chloride (Alchem Group, Toruń, cPoland) were dissolved in 10 mL of water and added to the main synthesis mixture. Next, 24.4 g of adipic acid was dissolved in 200 mL of isopropyl alcohol (Alchem Group, Toruń, Poland). This mixture was added dropwise for 2.5 h. The reaction mixture was kept at 70 °C for additional 1 h. Then, 500 mL of benzene (Alchem Group, Toruń, Poland) were added, and the reaction mass was transferred to the dividing funnel. After demixing, the bottom aqueous layer was removed and the upper layer was washed out by water till the catalyst was separated. ED-24AK was purified using vacuum distillation of the solvents. A total 71.8 g of the product was obtained (yield 85.7%). Physical properties of the resin are presented in a previous paper [20].

NBR was crosslinked with sulfur system containing stearic acid, zinc oxide (ZnO), sulfur, and dibenzothiazyl disulfide MBTS (DM). All components of the curing system were purchased from Sigma Aldrich (Taufkirchen, Germany). The composition of rubber mixes are given in Table 1. Moreover, a reference sample was also made, containing no resin added.

### 2.2. Sample Preparation

The rubber mixes were made by means of a 400 mm David Bridge laboratory two-rolls mill (Dudley, UK), operating with a friction of 1.1 and temperature not exceeding 40 °C. The following mixing regime was applied:

1. Mastication of rubber—2 min;
2. Admixing of resin—2 min;
3. Addition and mixing of crosslinking system—1 min, making the total time of mixing ca. 5 min.

Rubber samples were press-molded under 160 °C, during t_90_ time, determined vulcametrically with a MDR 2000 instrument (Alpha Tech., Hudson, Ohio, USA) according to PN–ISO 3417.

### 2.3. Kinetics of Crosslinking

The curing process of rubber mixes was investigated using an Alpha MDR 2000 (Hudson, OH, USA) rheometer, operating with an oscillation frequency of 1.70 Hz and an oscillation angle of 3°, at a temperature of 160 °C, according to PN-ISO 3417. Based on the experimental data, curing parameters were determined, namely optimum vulcanization time (t_90_), scorch time (t_05_), max. (MH), and min. (ML) torque. The conventional cure rate index (CRI) of the rubber compounds studied was calculated according to the equation [28,29]

CRI = 100/(t_90_ − t_05_)

where t_90_—optimum vulcanization time; t_05_—scorch time.

### 2.4. Crosslink Density

Crosslink density of rubber vulcanizates (ν) was determined by applying the procedure of equilibrium swelling in toluene [30], according to PN-ISO 1817: 2001. Samples cut from 2 mm thick plates, weighing 30–50 mg, were exposed to the solvent for 72 h, subjected to decanting and replenishing every 24 h. After drying on filter paper, the samples were weighed again and then dried to constant weight at 60 °C in a laboratory oven (MRC Laboratory Instruments, Essex, UK). The results of equilibrium swelling were applied for calculations of their crosslink density with calculated values of polymer—toluene Flory–Huggins′ parameters [31] using the Flory–Rehner formula [32].

Additional treatment with ammonia consisted of swelling the vulcanized samples in toluene under ammonia-saturated vapor in a desiccator at room temperature for a period of 48 h (ν_A_) to recognize whether the crosslinked polymers contain any non-covalent crosslinks [33,34]. The concentration of the possible specific links was estimated from the difference between crosslink density determined by the equilibrium swelling of the vulcanizates in toluene (ν) and ν_A_ [35,36].

### 2.5. FT-IR Analysis

Infrared FT analysis of epoxy resins and modified rubbers was carried out with a Nicolet 8700 (Thermo Scientific, Waltham, MA, USA) spectrometer, operating with the resolution of 4 cm^−1^ in the wavelength range 400–4000 cm^−1^.

The spectra were analyzed for changes accompanying resin or rubber modification. The following absorption bands, characteristic NBR:

- 2950, 2850, 1450, 1000, 900 cm^−1^ originated from C-H groups;
- 1550 cm^−1^ -C=C- from butadiene;
- 750 cm^−1^ C-H bonds in -CH=CH- butadiene monomer units;
- 2250 cm^−1^ -CN groups from acrylonitrile monomer units.

and epoxy resins:

- 1050 cm^−1^ originating from C-O bonds;
- 900 cm^−1^ typical for the presence of an epoxy ring;
- 1700, 1450 and 750 cm^−1^ characteristic for adipic acid;
- 550 cm^−1^ derived both from a functional group X in the para position in C_6_H_4_X_2_ of Epidian and from adipic acid were assigned and changes to their intensity and position discussed.

### 2.6. Mechanical Properties

Tensile strength (TS), moduli under elongation (SE), and elongation at the break of crosslinked rubber samples were determined with an universal mechanical testing machine Zwick 1345 (ZwickRoell GmbH & Co. KG, Ulm, Germany), according to PN-ISO 37, using 4 mm width and 1 mm thick specimens. Hardness of the samples was determined with a Zwick Shore A durometer 3130 (Zwick-Roell GmbH & Co. KG, Ulm, Germany), according to EN ISO 868.

### 2.7. Surface Energy

The sessile drop method (droplet volume set for: 1 µL) was utilized for contact angle measurements. An OCA 15EC goniometer (DataPhysics Instruments GmbH, Filderstadt, Germany) equipped with a single direct dosing system (0.01–1 mL B. Braun syringe, Melsungen, Germany) was employed. Two liquids differing in polarity—distilled water and 1,4-dioodomethane—were used under ambient conditions. Before the measurement, surfaces of rubber vulcanizates were cleaned with acetone. Surface free energy was calculated with the Owens–Wendt–Rabel–Kaelble (OWRK) method [37].

### 2.8. Adhesion Measurements

**Figure 2:**
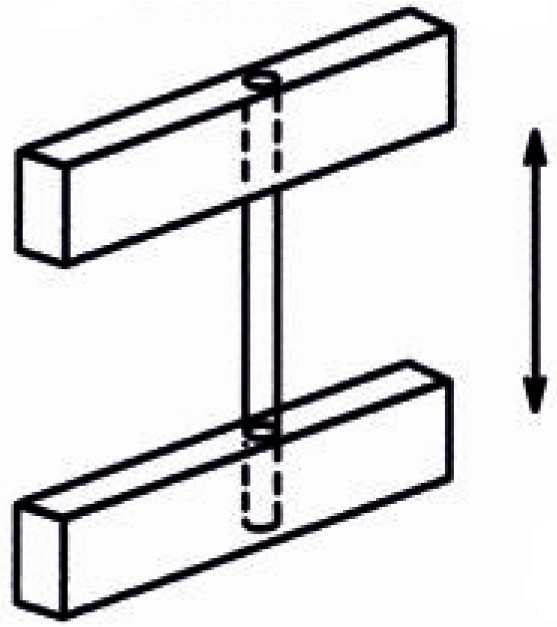
Illustration of the “H”-type specimen. A glass fiber was fixed in two rubber plates during sample preparation. Arrow shows direction of the sample elongation.

The pull-out tests of glass fibers (ø = 1 mm) from rubber samples were performed according to PN-81/C-04267 (the so-called method of the “H” type specimen; see Figure 2) using an universal mechanical testing machine Zwick 1345 (ZwickRoell GmbH & Co. KG, Ulm, Germany) operating with a linear speed of 50 mm/min.

### 2.9. Friction Measurement

The tribological characteristics of the vulcanizates were obtained using a T-05 tribometer with a block-on-ring friction pair (ITeE-PIB, Radom, Poland). The tests were conducted under normal conditions. A block made of stainless steel, loaded with a normal force P = 1 N, was pressed against a 40 mm of diameter rubber ring made of tested material, rotating with 70 rpm. The experimental data were acquired using a SPIDER 8 (Hottinger Messtechnik, Darmstadt, Germany) electronic system. The median value of friction force from 3 test runs, collected after 30 min required for friction stabilization, was adopted as the friction force of material. The friction coefficient (µ) was calculated on the basis of the Amontous formula [38].

## 3. Results and Discussion

### 3.1. Kinetics of Crosslinking

**Figure 3:**
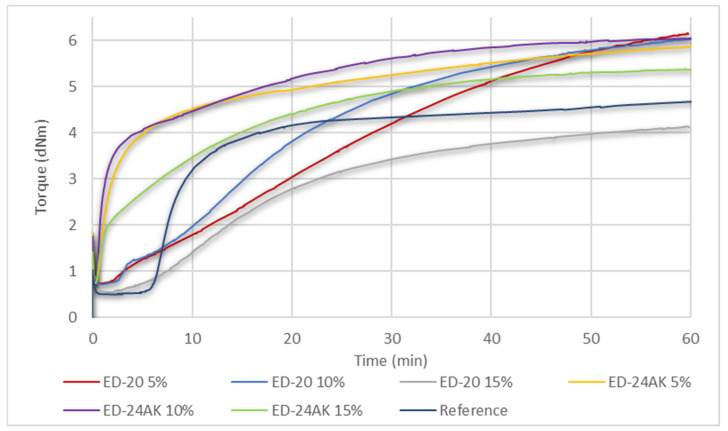
Vulcanization kinetics of rubber vulcanizates studied.

Crosslinking kinetics of rubber vulcanizates studied are presented in Figure 3, and their curing parameters are shown in Table 2.

The addition of epoxy resin practically does not influence the rheology of rubber mixes—similar values of ML were presented no matter the kind or the amount of resin added. All the resin tested exhibited the ability to crosslink NBR; however, generally, the reaction was faster—t_05_ and t_90_ were shorter for rubber samples modified with ED-24AK in comparison to ED-20, but much longer for both materials when related to unmodified NBR. The increase in torque is similar in both cases; however, the amount of resin added above 5 wt.%, no matter the increased vulcanization time, also adversely affected the torque increase (ΔM), which is especially visible for the addition of ED-20.

The addition of epoxy resin made the vulcanization time of rubber longer, but only for ED-20 addition, which is opposite to the results for the addition of its modified analogue. The higher the percentage of ED-20 resin, the higher the t_05_, which is the opposite to the results for ED-24AK addition. The ED-24AK 10% sample exhibited the highest CRI value among the samples studied. Addition of ED-20, compared to the reference, decreased CRI, but the addition of ED-24AK increased this parameter. The CRI increased with increasing resin addition. Moreover, it is higher for ED-24AK composites, indicating its contribution to the crosslinking of the system (see Section 3.4).

### 3.2. Crosslink Density

**Figure 4:**
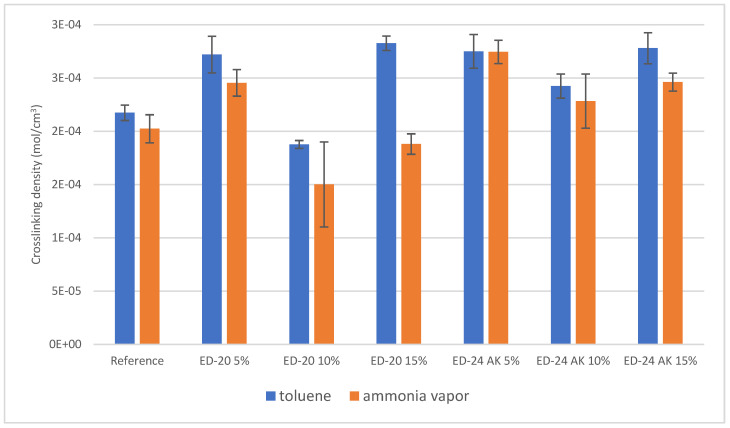
Influence of resin addition on the crosslink density of NBR (nitrile butadiene rubber) vulcanizates.

The results of crosslink density for the rubber vulcanizates studied are presented in Figure 4.

Based on the above results, it can be concluded that the addition of resins in the amounts of 5 wt.% and 15 wt.% increases the crosslink density, while the addition of 10 wt.% reduces it. The same thing occurs with both resins, which may be related to the morphology of the system. Too little or too much resin content has a negative effect on the degree of its dispersion in the rubber matrix. Paradoxically, it may have a positive effect on the degree of the rubber crosslinking (Figure 4) due to aggregated resin particles not interfering with crosslinking of the rubber phase. The observed effect of lowering the density of the rubber matrix was lower in the case of the addition of the resin modified with adipic acid, which showed a much greater tendency to internal and external crosslinking (Section 3.4), contributing to the total crosslinking of the system.

It can also be seen that, for both kinds of resins added in higher amounts, we deal with a slight share of bonds that break down under the influence of ammonia vapors, pointing to the creation of ionic crosslinks involving possible interactions between nitrile groups of NBR and polar groups of resins.

### 3.3. FT-IR (Fourier-Transform Infrared Spectroscopy) Analysis

**Figure 5:**
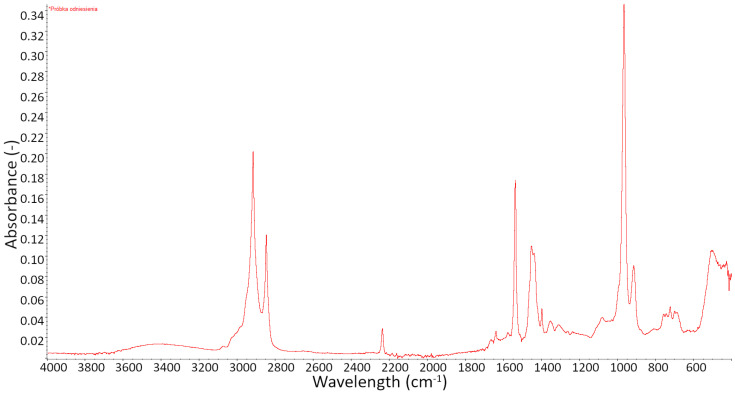
FT-IR spectrum of the reference.

In the NBR spectrum (Figure 5), one can observe peaks typical for NBR rubber and a small peak around 3400 cm^−1^ belonging to hydroxyl groups, probably originating from a small degree of rubber oxidation. The peaks around 2950, 2850, 1450, 1000, and 900 cm^−1^ belong to different types of C-H groups. In 2250 cm^−1^, we can observe a peak typical for -CN groups from acrylonitrile and in 1550 cm^−1^ -C=C- from butadiene. The peak around 750 cm^−1^ also comes from butadiene, which belongs to the C-H bonds in -CH=CH-.

**Figure 6:**
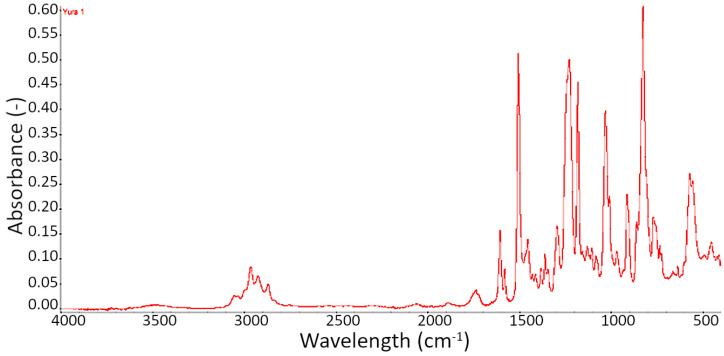
FT-IR spectrum of ED-20 resin.

Figure 6 presents the infrared spectrum of ED-20 epoxy resin (Epidian 5), containing absorption bands at 1050 cm^−1^ originating from C-O bond’s, 900 cm^−1^—typical for the presence of an epoxy ring, and two peaks in the wavelength region 500–550 cm^−1^, which most likely derives from C_6_H_4_X_2_, where X is a functional group in the para position [36] and from adipic acid, as evidenced by the high height of the peak. Apart the above, there are also peaks present at 3400 cm^−1^, belonging to -OH resin groups; 1500 cm^−1^, originating from the C-C bonds of the resin aromatic groups; 1250 cm^−1^ from C-O bonds of the aromatic ring; 1200 cm^−1^ from aliphatic C-O, 2950; 2850 and 800 cm^−1^—from C-H bonds of various types; and 1600 and 1450 cm^−1^ from the C-C bond in the aromatic ring.

**Figure 7:**
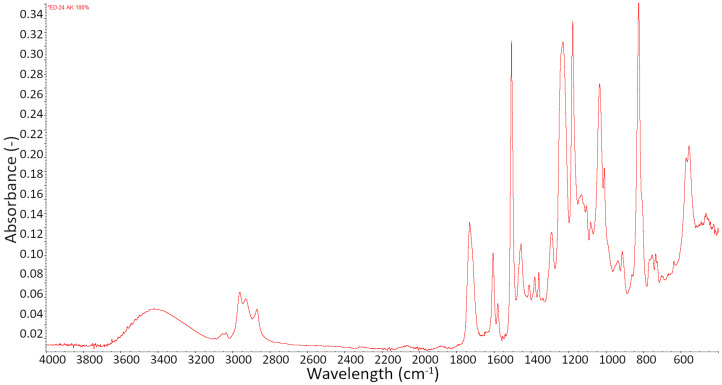
FT-IR spectrum of ED-24 AK resin.

In this spectrum belonging to ED-24 AK modified resin (Figure 7), one can observe both peaks originating from ED-20 (Epidian 5) and those typical for adipic acid at around 1700, 1450, 750, and 550 cm^−1^.

**Figure 8:**
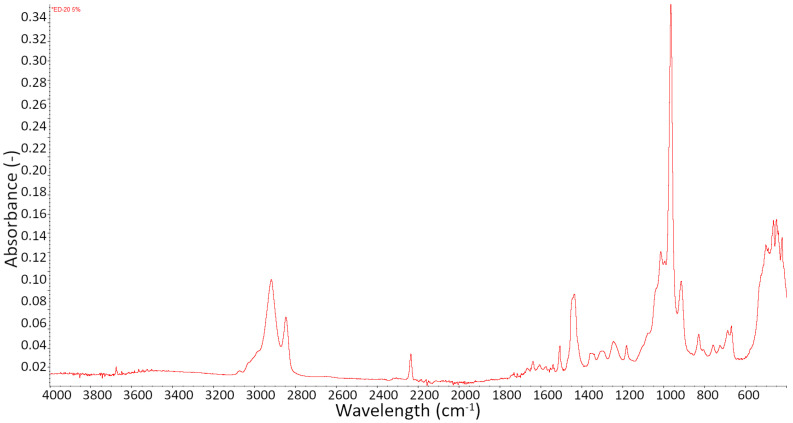
FT-IR spectrum of NBR modified with 5 wt.% of ED-20 after vulcanization.

In the spectrum of NBR modified with 5% of ED-20 (Figure 8), typical absorption peaks for NBR rubber and the disappearance of the peak around 3400 cm^−1^ (-OH groups) can be observed. The peak at 1550 cm^−1^ -C=C- from butadiene decreases significantly, which indicates the disappearance of this type of bonds in NBR. Typical Epidian 5 peaks appear 1050 cm^−1^ from the C-O bond and 900 cm^−1^ peak for the presence of an epoxy ring and increased peak at 550 cm^−1^, which confirms the resin content.

**Figure 9:**
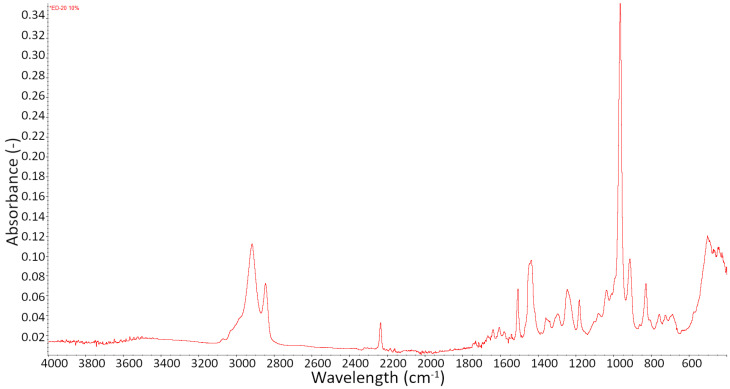
FT-IR spectrum of NBR modified with 10 wt.% ED-20 after vulcanization.

Analogous absorption peaks are present in the spectrum of ED-20 10% (Figure 9), except that the resin-derived peaks become larger, so that a few more typical for ED-20 ones come to the significant level. They are 3400 cm^−1^ belonging to the -OH group in the resin, 1500 cm^−1^ from the C-C bond of the aromatic groups of the resin, 1250 cm^−1^ from the C-O bond of the aromatic ring, and 1200 cm^−1^ from aliphatic C-O groups. There is also a significant increase in the peak typical for the content of epoxy groups.

**Figure 10:**
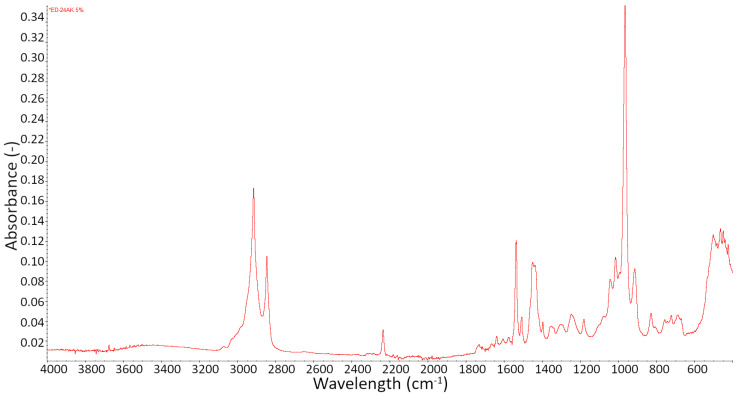
FT-IR spectrum of NBR modified with 5 wt.% ED-24AK after vulcanization.

In the case of the sample containing 15% of the resin, one can witness another increase in the intensity of the peaks coming from the resin. In the FT-IR spectrum of ED-24 AK sample, one can see relatively high peaks from NBR rubber and small peaks from a resin (Figure 10).

**Figure 11:**
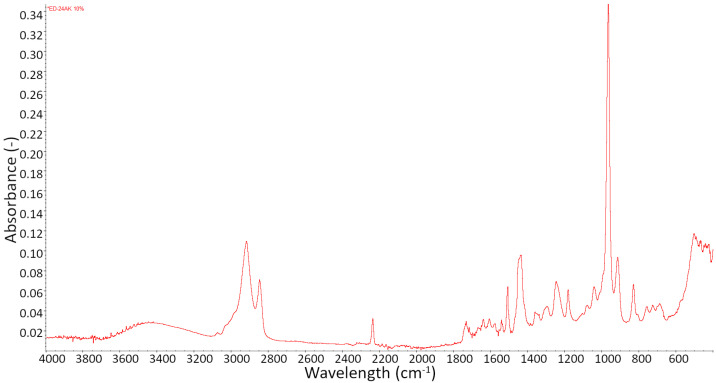
FT-IR spectrum of NBR modified with 10 wt.% ED-24AK after vulcanization.

As in the case of samples with unmodified resin, along with an increase in the ED-24 AK content, we observe a slight decrease in the rubber peaks and an increase in the resin peaks (Figure 11). The peaks from adipic acid are slightly strengthened, although this is a smaller increase than in the case of the peaks from ED-20, which again confirms that the acid is there but its content in the resin is small.

In the case of the sample with 15% resin, a further increase in the intensity of the peaks coming from the resin can be observed, which proves its higher content.

### 3.4. Mechanism of Crosslinking

The mechanism of NBR crosslinking with sulfur and epoxy resins was examined using IR spectra represented in Figure 5, Figure 6, Figure 7, Figure 8, Figure 9, Figure 10 and Figure 11.

One can see from Figure 5 that NBR contains double bond C=C (1550 cm^−1^) and CN group (2250 cm^−1^). ED-20 epoxy resin (Figure 6) contains only one functional group—the reactive epoxy group (910 cm^−1^). The free epoxy group (910 cm^−1^) and hydroxy group (3450 cm^−1^) indicate the presence of carboxy group, as well as secondary hydroxy group (950 cm^−1^), in the structure of ED-24 AK (Figure 7).

**Scheme 1:**
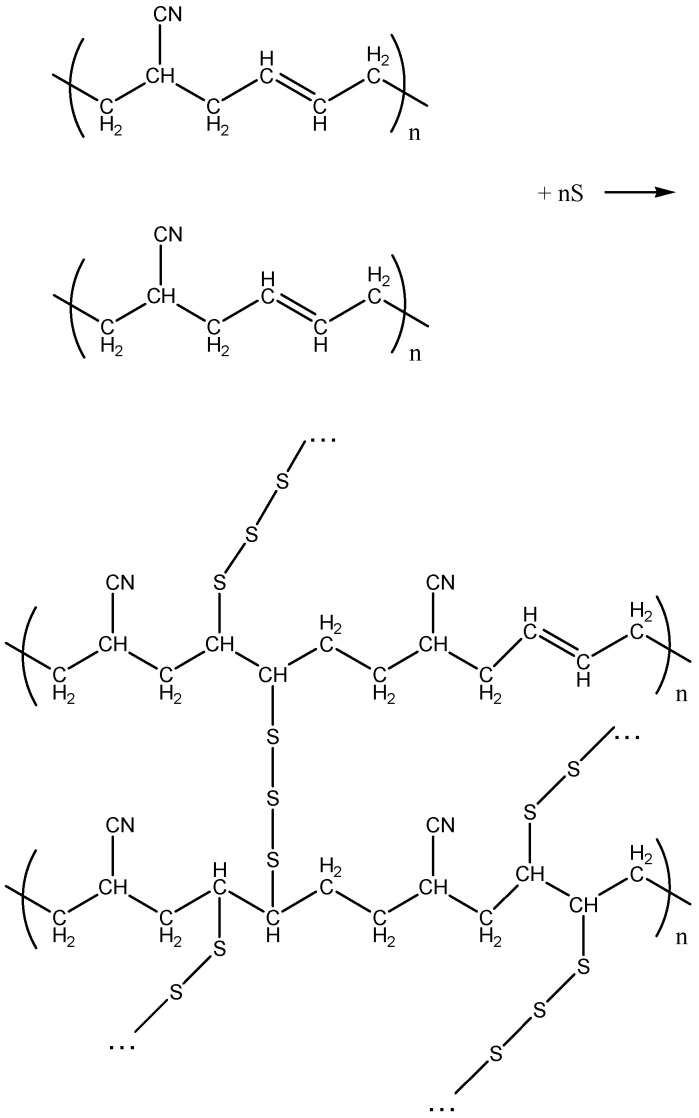
Crosslinking of NBR with sulfur.

**Scheme 2:**
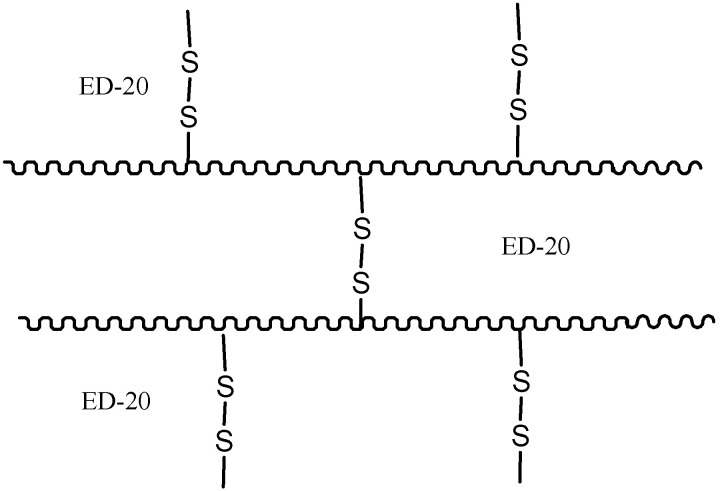
Structure of the system contained NBR crosslinked with sulfur and epoxy dian resin.

During crosslinking of the system consisting of rubber, sulfur, and various amounts of ED-20 (Figure 8 and Figure 9), the decrease in intensity of the absorption band at 1550 cm^−1^ is observed, whereas the bands at 910 cm^−1^ and 2250 cm^−1^ remain unchanged. Therefore, this prompts the conclusion that because of the lack of functional groups capable of reacting with ED-20 epoxy groups, the rubber crosslinking occurs only due to the interaction of molecular sulfur with regard to double bonds of the rubber. The crosslinked structure is formed according to Scheme 1:

ED-20 molecules exist inside the crosslinked network; i.e., the structure shown in Scheme 2 is formed.

Such a position of ED-20 molecules affects rubber properties, which are represented in Table 3 and Table 4 (see the next chapter). The properties depend on the amount of ED-20 introduced to the mixture.

**Scheme 3:**
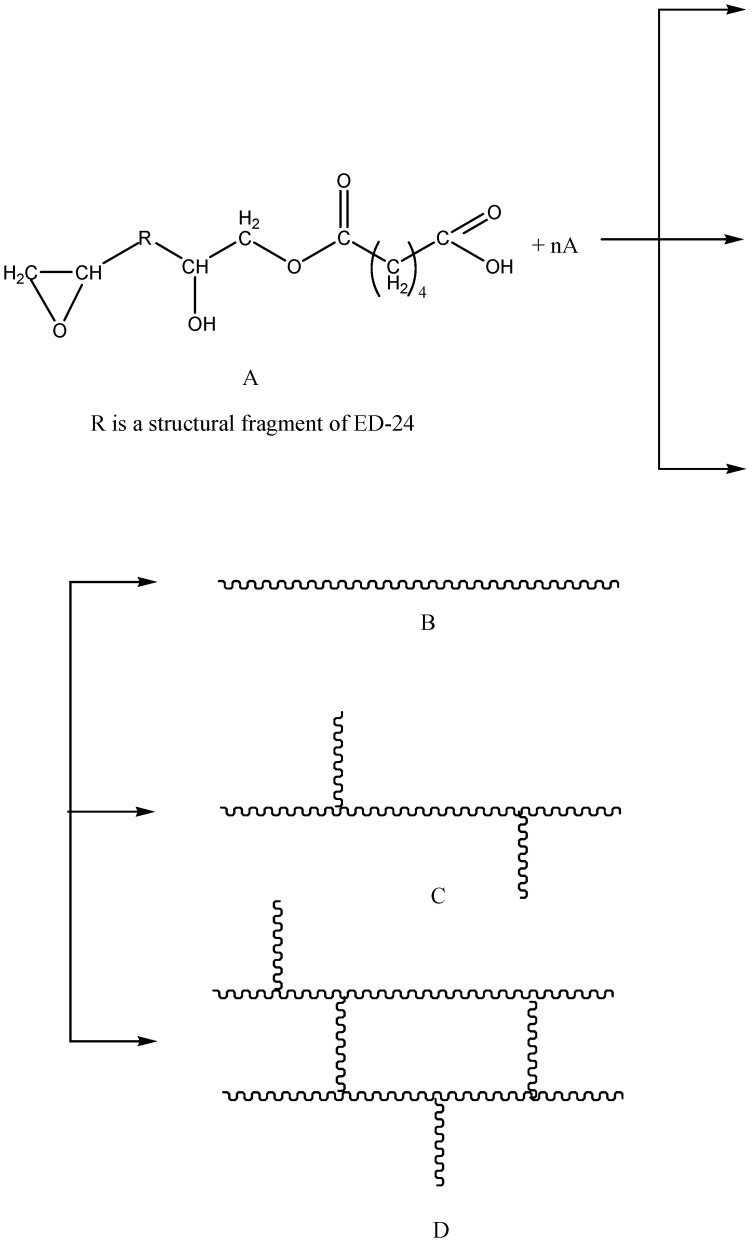
Various possibilities of chemical reactions taking place in the system of NBR containing adipic acid modified epoxy dian resin.

Another situation is observed when ED-24 AK is used. This resin contains functional groups that are different by nature, which are unable to react with functional groups of the rubber. Under conditions of rubber vulcanization (the temperature of 160 °C), the functional groups can react according to Scheme 3.

In the first option (Scheme 3), epoxy groups of ED-24 AK react with carboxy group of another molecule, and a linear product (B) is formed.

In the second option, functional groups of ED-24 AK (epoxy or carboxy group) react with hydroxy groups of another molecule, and grafted products (C) are formed.

In the third option, functional groups of ED-24 AK (epoxy, carboxy, and hydroxy groups) react between each other, and a partially crosslinked structure (D) is formed.

**Scheme 4:**
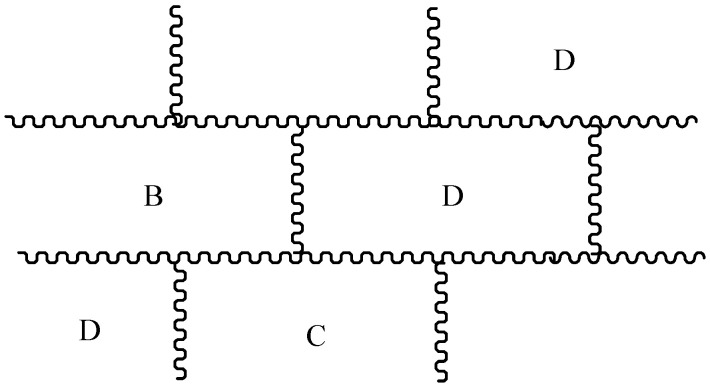
Structure of the system containing NBR crosslinked with sulfur and adipic acid modified epoxy dian resin.

Therefore, the structure shown in Scheme 4 is formed during rubber curing in the presence of ED-24 AK.

In the case of (B) and (C) formation, a vulcanized rubber with non-crosslinked molecules of ED-24 AK will be formed.

In the third option, a penetrating network is formed as a result of rubber curing with sulfur.

To summarize the above, when NBR is crosslinked with sulfur, ED-20 and ED-24 AK resins do not enter the structure of the vulcanized rubber but act as additives. ED-20 acts without changes in its structure, whereas ED-24 AK is a partially crosslinked additive.

### 3.5. Mechanical Properties

Mechanical properties of NBR modified with the epoxy dian resins are summarized in Table 3.

Addition of any resin makes TS parameter higher. The results obtained demonstrate that only addition of ED-24 AK in the highest amount has a significant strengthening effect on NBR, as demonstrated by TS and Eb data. The addition of ED-20, no matter the amount studied, is less effective in mechanical strength in terms of NBR improvement, which stays in agreement with previous crosslink density data. In the case of ED-20 resin, its addition reduces the hardness of the finished composite (the higher its content, the more there is). For the ED-24AK, however, we observe the opposite dependency, which can be ascribed to strong effect of internal crosslinking (Section 3.4), contributing to mechanical strength parameters and hardness of the system.

### 3.6. Contact Angle and Surface Energy

**Figure 12:**
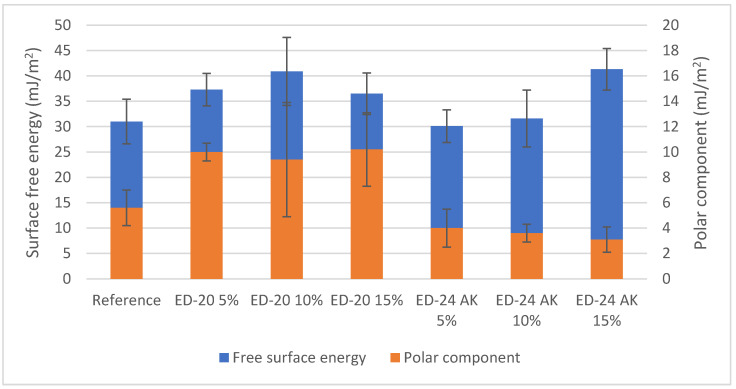
Surface energy and its components of the samples studied.

The distribution of free surface energy components for samples (Figure 12) does not show any strong relationship between the addition of resins and free surface energy. However, it can be seen that the addition of ED-24 AK resin slightly reduces free surface energy. In the dispersion component, it can be seen that the addition of the modified resin with an increasing amount increases the proportion of the dispersion component. In the case of unmodified resin, there is no such relationship. In the case of the polar component, no relationship can be seen for the unmodified resin. The modified resin, along with an increase in its amount in the polymer, slightly reduces the proportion of the polar component.

### 3.7. Adhesion Measurements

Adhesion of NBR modified with epoxy dian resins to glass fibers is presented in Table 4.

Addition of any resin promotes rubber adhesion to glass fiber. ED-20 resin promotes NBR adhesion to glass fibers to a higher extent in comparison to the ED-24 AK one, probably because it is not involved in crosslinking. There is practically no influence of the ED-20 content on the adhesion, but there was a rather surprisingly a drop for the rubber sample containing 10% of the adipic acid modified resin, which agrees with the crosslink density data (Figure 4).

### 3.8. Friction Characteristics

**Figure 13:**
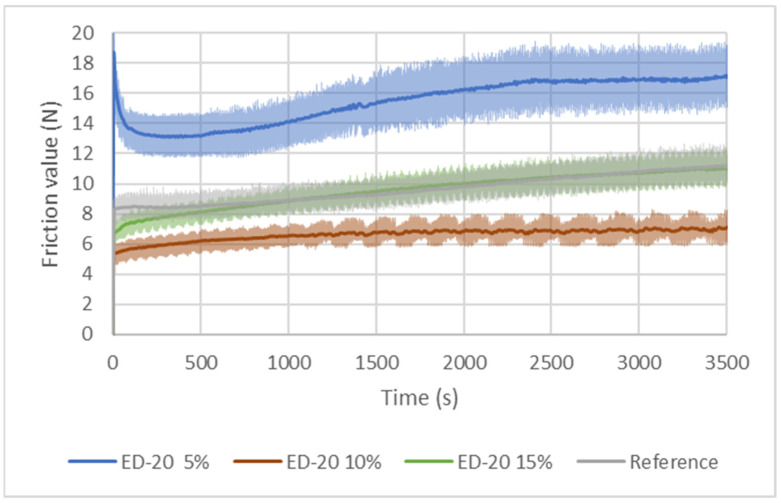
Friction characteristics of NBR modified with the addition of ED-20.

**Figure 14:**
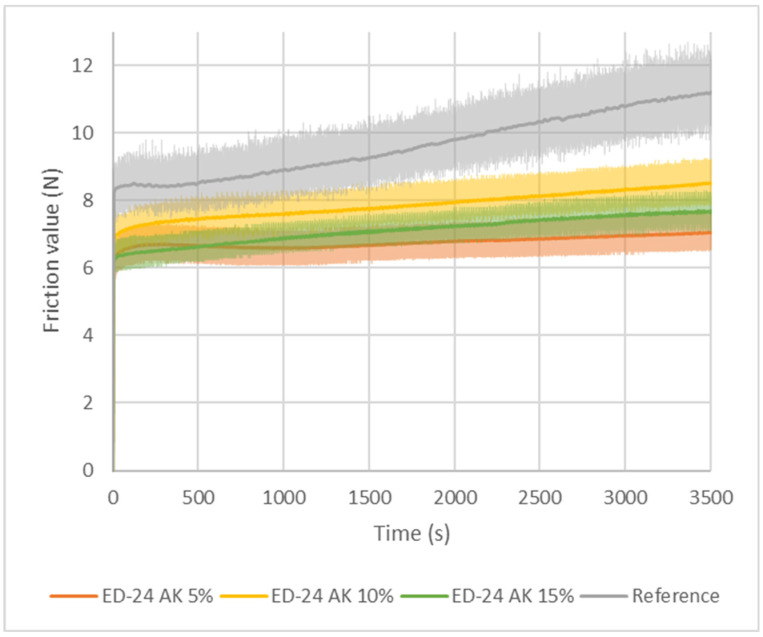
Friction characteristics of NBR modified with the addition of ED-24 AK.

Friction characteristics of the epoxy dian modified NBR samples are presented in Figure 13 and Figure 14.

The addition of unmodified resin (ED-20) in an amount of 10% makes the reduction of the friction force for NBR vulcanizate the most significantly. The addition of 15% practically does not influence the level of frictional force. Despite a possible lubricating effect presented by the resin addition, its plastifying effect makes the reduction of NBR friction force less visible in comparison to the admixing of its adipic acid modified form (ED-24 AK). Modification of the resin with adipic acid reduces the friction force of NBR when it is added at 5% and increases the contents at 10% and 15%. It seems likely that crosslink density and the related stiffness of rubber vulcanizates are responsible for the friction force reduction. This explanation agrees with the data for the crosslink density (Figure 4) and mechanical properties (Table 3).

## 4. Summary and Conclusions

Based on the results obtained, it can be concluded that

1. FTIR analysis reveals that absorption peaks originated from both resins and rubber; however, characteristic peaks of ED-20 are more visible in comparison to ED-24 AK, which can be associated with the active participation in crosslinking of rubber only for the latter.
2. The addition of adipic acid modified dian resin increases the crosslink density of NBR (of both covalent and ionic characters).
3. The effect of NBR modification by epoxy dian resins is likely to be related to the morphology of the system. Too little or too much resin content has a negative effect on the degree of its dispersion in the rubber matrix. The aggregated resin particles do not interfere with the crosslinking of rubber phase. The observed effect of lowering the density of the rubber matrix is lower in the case of the addition of ED-24 AK, which shows a much greater tendency for internal and external crosslinking, contributing to the total crosslinking of the system.
4. The above conclusions explain the findings that the addition of ED-20 is more efficient in terms of rubber adhesion to glass fibers, whereas ED-24AK is better according to its mechanical properties (TS, moduli in extension and hardness).
5. The addition of ED-20 results in a higher surface energy of NBR in comparison to ED-24 AK resin. Despite higher polarity of the modified epoxy resin, due to its participation in rubber crosslinking, the NBR vulcanizates modified with ED-24 AK show lower surface polarity than those for which ED-20 was used. This better explains the adhesion of NBR modified with unmodified epoxy resin to glass fibers.
6. The resin addition in the amount of 5 phr has the greatest influence on the friction force of NBR vulcanizates (increasing for ED-20; decreasing for ED-24AK), due to different mechanism of their action involved, i.e., plastification for the former and hardening for the latter. The optimal amount of the tested resin modifiers from the point of view of reducing NBR friction, regardless of their type, is 10 phr. In the case of ED-20, this can be explained by the best lubricating effect, with moderate plasticization of vulcanizates. On the other hand, in the case of the ED-24 AK, we deal with an optimal combination of a greater stiffness of the vulcanizate with a reduced polarity of its surface.

Summary of the results obtained is presented in Table 5.

Depending on the desired properties of the final composite, NBR can be modified either with ED-20 resin or with its adipic acid modified analogue (ED-24 AK). The coupling makes it possible to crosslink the rubber and to tailor its mechanical properties, namely adhesion and friction.

## Figures and Tables

**Table 1:** Composition of the rubber mixes studied (phr).

Components	5% of Resin		10% of Resin		15% of Resin	
	(phr)	(g)	(phr)	(g)	(phr)	(g)
Nancar 2865 (NBR)	100.0	60.0	100.0	55.0	100.0	50.0
Stearic acid	1.0	0.6	1.0	0.55	1.0	0.5
ZnO	5.0	3.0	5.0	2.75	5.0	2.5
DM (MBTS)	1.0	0.6	1.0	0.55	1.0	0.5
Sulfur	1.5	0.9	1.5	0.83	1.5	0.75
ED-20/ED-24AK	5.0	3.0	10.0	5.5	15.0	7.5

**Table 2:** Data collected from the vulcanization kinetics study.

	Ref.	ED-20 5%	ED-20 10%	ED-20 15%	ED-24AK 5%	ED-24AK 10%	ED-24AK 15%
ML (dNM)	0.5	0.7	0.6	0.6	0.8	0.8	0.7
MH (dNM)	4.9	6.2	5.5	4.2	5.9	5.6	5.4
ΔM (dNM)	4.4	5. 4	4.9	3.6	5.1	4.8	4.7
t_05_ (min)	6.1	3.2	4.0	4.9	0.7	0.6	0.5
t_90_ (min)	38.4	47.4	42.4	40.7	34.2	27.1	30.9
CRI (100%/min)	3.1	2.3	2.6	2.8	3.0	3.8	3.3

**Table 3:** Mechanical characterization of the rubber samples studied.

Parameter	Ref.	ED-20			ED-24 AK		
	-	5%	10%	15%	5%	10%	15%
TS (MPa)	2.09	3.31	2.61	3.21	4.91	4.41	7.13
Eb (%)	646	270	695	560	513	526	339
SE100 (MPa)	0.85	0.81	0.63	0.69	0.76	0.91	0.86
SE200 (MPa)	1.06	1.03	0.93	1.01	1.08	1.6	1.5
SE300 (MPa)	1.22	1.34	1.24	1.33	1.51	2.4	2.4
Hardness (°ShA)	38.1	37.2	35.5	32.1	38.5	41.6	40.4

**Table 4:** Properties of adhesion to glass fibers of the rubber samples studied.

Parameter	Ref.	ED-20			ED-24 AK		
		5%	10%	15%	5%	10%	15%
Adhesion (N):							
Average	18.5	27.7	24.5	26.5	22.1	15.6	23
Std deviation	3.8	4.5	4.1	5.3	5.4	3.7	3.7

**Table 5:** Relative change in selected properties of NBR vulcanizates as a result of their modification with epoxy dian resins.

	Sample	ED-20 5%	ED-20 10%	ED-20 15%	ED-24 AK 5%	ED-24 AK 10%	ED-24 AK 15%
Property Tested							
Curing time (min)		↑ (+23%)	↑ (+10%)	↑ (+6%)	↓ (−16%)	↓ (−29%)	↓ (−20%)
Crosslink density (mol/cm^3^)		↑ (+25%)	↓ (−14%)	↑ (+30%)	↑ (+27%)	↑ (+12%)	↑ (+28%)
Tensile Strength (MPa)		↑ (+58%)	↑ (+25%)	↑ (+54%)	↑ (+135%)	↑ (+111%)	↑ (+241%)
Elongation at break (%)		↓ (−58%)	↑ (+8%)	↓ (−13%)	↓ (−21%)	↓ (−19%)	↓ (−48%)
Hardness (°Sh A)		↓ (−2%)	↓ (−7%)	↓ (−16%)	↑ (+1%)	↑ (+9%)	↑ (+6%)
Surface energy (mJ/m^2^)		↑ (+19%)	↑ (+32%)	↑ (+16%)	↓ (−3%)	↓ (+3%)	↑ (+35%)
Adhesion (N)		↑ (+50%)	↑ (+32%)	↑ (+43%)	↑ (+19%)	↓ (−16%)	↑ (+24%)
Friction (N)		↑ (+113%)	↓ (−25%)	↓ (+25%)	↓ (−12%)	↓ (0%)	↓ (−12%)

## Data Availability

The data presented in this manuscript are available on request from the corresponding authors.
